# The Concept of a Quantum Edge Simulator: Edge Computing and Sensing in the Quantum Era

**DOI:** 10.3390/s23010115

**Published:** 2022-12-23

**Authors:** Ali Passian, Gilles Buchs, Christopher M. Seck, Alberto M. Marino, Nicholas A. Peters

**Affiliations:** Quantum Information Science Section, Oak Ridge National Laboratory, Oak Ridge, TN 37831, USA

**Keywords:** quantum sensing, sensors, quantum sensors, edge computing, quantum processors, quantum communication, quantum network, quantum internet

## Abstract

Sensors, enabling observations across vast spatial, spectral, and temporal scales, are major data generators for information technology (IT). Processing, storing, and communicating this ever-growing amount of data pose challenges for the current IT infrastructure. Edge computing—an emerging paradigm to overcome the shortcomings of cloud-based computing—could address these challenges. Furthermore, emerging technologies such as quantum computing, quantum sensing, and quantum communications have the potential to fill the performance gaps left by their classical counterparts. Here, we present the concept of an *edge quantum computing (EQC) simulator*—a platform for designing the next generation of edge computing applications. An EQC simulator is envisioned to integrate elements from both quantum technologies and edge computing to allow studies of quantum edge applications. The presented concept is motivated by the increasing demand for more sensitive and precise sensors that can operate faster at lower power consumption, generating both larger and denser datasets. These demands may be fulfilled with edge quantum sensor networks. Envisioning the EQC era, we present our view on how such a scenario may be amenable to quantification and design. Given the cost and complexity of quantum systems, constructing physical prototypes to explore design and optimization spaces is not sustainable, necessitating EQC infrastructure and component simulators to aid in co-design. We discuss what such a simulator may entail and possible use cases that invoke quantum computing at the edge integrated with new sensor infrastructures.

## 1. Introduction

With the announcement of the new supercomputer *Frontier*, we have entered the exascale computing age [1]. Novel paradigms of computing and communications technologies are being explored to enable the wider use of IT. These include federated computing [2], edge computing [3,4], quantum computing [5], quantum networking and the envisioned quantum internet [6,7,8], among others, all of which promise exciting new capabilities and broader access to number crunching, data processing, and data use. To generate computing results, federated computing [9] brings the computation to where the data resides, e.g., in a medical device memory for which patient privacy measures related to personally identifiable information (PII) and protected health information (PHI) may exclude the transfer of the data to computing resources [10]. Edge classical computing (ECC) brings processing power (e.g., in the form of an edge server) closer to where the data are generated, even possibly closely integrated with the very sensor acquiring the data. On the other hand, quantum computing promises to grossly outperform even the best classical computers for certain classes of problems [5] and could become a coprocessing element of any future computing infrastructure [11]. Here, we consider quantum computing at the edge integrated with classical and quantum sensors. The same quantum resources that can be used to perform quantum computation can also be configured as quantum sensors [12], potentially improved through quantum algorithms [13,14,15], to form powerful new edge quantum computing (EQC) infrastructures.

The ever-increasing demand for real-world IT applications necessitates the use of various simulators. To mimic the function and operation of a realistic use case, a simulator requires models that can predict the response or behavior of the increasingly complex and sophisticated subsystems and components that make up the use case. As a specific application that may highlight the need for an EQC in the near future, especially one that would be challenging to accomplish without a simulator, we note the current work on networks of entangled atomic clocks to enhance the precision of time and frequency measurements for a large number of applications, both from a fundamental scientific perspective, such as dark matter detection, and for more practical applications, such as positioning and navigation [16,17]. Here, in introducing the concept of an EQC simulator, we pose certain relevant questions. While addressing these questions will require massive future research, these early discussions may help pave the way toward a plausible roadmap.

With the advances in high-performance computing (HPC) and the growth in the number of networked devices, the need for simulating [18] a variety of network-location-dependent compute scenarios is increasing. Design and optimization of the data-generating intelligent sensors and actuators that are distributed over the network, i.e., internet-of-things (IoT) devices, require variation and integration of many parameters that characterize at least time, space, load, bandwidth, and energy dependencies of the final system. ECC is a data processing paradigm aiming at countering the current and projected bottlenecks of cloud-based computing [3,4]. An ECC device attempts to process all data on local computing and communications infrastructure and, if necessary, establishes a communication link to a data center for additional/further processing. Therefore, both latency and memory bottlenecks of the current centralized cloud-based paradigm may be addressed. The recent development of processors for ECC applications [19] helps realize the promise of ECC. Importantly, as a new information technology, ECC addresses the challenges associated with the growing number of mobile and stationary devices that generate, displace, and process data. Therefore, the ability to simulate ECC scenarios is increasingly needed to improve use case development and optimization. To achieve this goal, prototyping ECC schemes with a dedicated simulator that natively considers ECC networks and computational resources is needed. As briefly reviewed below, the ECC simulators reported thus far, are all based on cloud-computing rather than edge-computing simulators [20].

IoT [21] is the realm of connectivity and continuity, in which a higher degree of freedom prevails for information. IoT thus aims to create seamless access, distribution, transport, and integration of information. CloudSim [22], iFogSim [23], and EdgeCloudSim [24,25] are software frameworks that simulate various IoT scenarios. Each package is created with specific attention to characteristics that differentiate the various computing domains. Cloud, cloudlet, fog, mist, and edge [21] refer to the various network locations/parameters where computing occurs. Information/data *I*, created at space–time coordinate (*x_c_*, *t_c_*), processed at location (*x_p_*, *t_p_*), and used at location (*x_u_*, *t_u_*), may be regarded as a multivariate function *I(x*, *t)*, and the parameter point *(c*, *p*, *u)* may thus designate the type of IT technology (edge, fog, etc.) invoked. For example, for ECC, $x_{p}≳x_{c}$ while for cloud computing, we may have $x_{p}\gg x_{c}$. Cloudsim, the main simulation toolkit for cloud computing environments, was developed specifically for cloud computing scenarios but was not designed for ECC simulation. Both iFogSim and EdgeCloudSim are Cloudsim-based simulators that include ECC simulation capabilities but were not originally designed or optimized for ECC simulation. For example, CloudSim does not provide communications models for wireless local area network (WLAN) and wide area network (WAN) connectivity, nor does it support physically mobile nodes such as mobile devices. Such differences can be articulated also for simulating an “edge processor” versus a “regular processor”, e.g., compare simulations of “edge-oriented” processors catered to power- and space-constrained edge locations [19] versus standard processors.

As envisioned here, EQC, on the other hand, is poised to take IT and its industries to a new level. However, given the cost and complexity of quantum systems, constructing physical prototypes to explore design and optimization spaces is currently challenging, and requires simulators to aid in nascent co-design efforts. Thus, simulators for quantum “edge systems” are needed. Nonetheless, a “quantum edge simulator” does not yet exist. Whether classical computing is invoked for simulating a quantum “edge system” or an all-quantum simulator (analog or digital) can be utilized to simulate the edge system needs to be explored; like simulators for HPC that may require simulation on HPC resources, EQC devices may require simulation on quantum resources. Such processes will benefit from efficient methods for simulating quantum systems on quantum computers [26]. Proposals to develop the first phase of quantum edge simulators to provide a framework for which quantum processors and algorithms can handle noisy data, data processing, error correction, optimization, communications, etc., are needed. Indeed, related paradigms are already being predicted, as discussed by Gill [27] in the context of serverless computing, blockchain technology, and quantum computing. Specifically, Gill advocates for a holistic computational platform encompassing devices at the service, management, and IoT layers [27].

**Figure 1:**
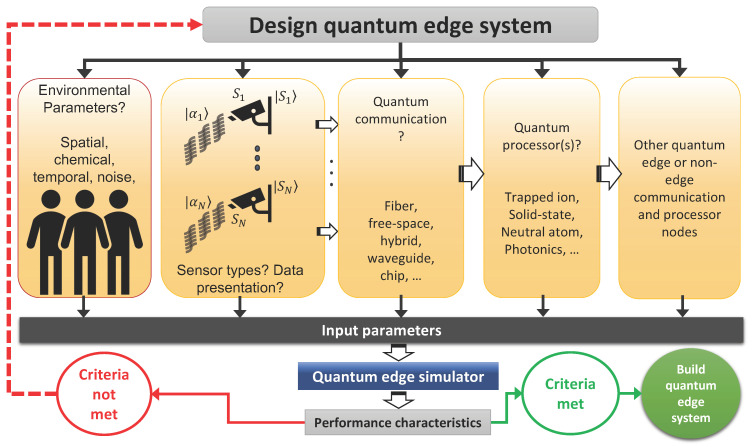
Depiction of a plausible standard framework for designing EQC systems and simulator requirements for effective design and evaluation. A well-established edge architecture requires many algorithms, models, parameter specifications, and optimizations for various types of processors, memory, network devices, noise, and other decoherence sources. Such a simulation would require a significant amount of processing power, processing speed, and the ability to co-process classical bits and qubits, a task that is likely outside the capabilities of a classical simulator.

What problems could such proposals address? Specifically, what requirements and protocols for quantum sensing, transduction, computing, communications, and networking need to be explored? For any initial phase, it would be reasonable to limit our consideration to the sensors and the processors only, that is, without considering the complexities of incorporating quantum and/or classical communications. With this basic design, it seems logical to simulate the simplest form of an EQC use case comprising a single sensor-processor pair. Consider a quantum-enhanced chemical sensor and a quantum processing unit (QPU), depicted in Figure 1. The sensor (i.e., a trace chemical detector) and its environment must be treated as an open quantum system. Noise, loss, and other dissipative processes as well as out-of-equilibrium processes will have to be considered, albeit at the cost of significant added complexity and intractability. The sensor’s dynamics may then be described via a master equation [28,29]. Once a measurement has occurred, the measurement data can be processed using the QPU. What QPU is more suitable for a given set of data so that the needed algorithms can be executed most efficiently will have to be addressed. Quantum processors are being developed by capitalizing on a variety of degrees of freedom in different physical settings including optical, atomic, electric, and magnetic systems [30]. Interfacing with these computing nodes and their classical layers is far from being implemented universally and requires consideration of many potential mismatches in signal pathways. For example, does the quantum sensor output a signal that is in the form of a quantum state or a classical quantity? This can further affect the efficacy of algorithms that use the data as input. Many quantum algorithms are touted to offer an advantage, notably speedup, over classical versions for the same task. If the input data are not naturally in a quantum state, then such advantages may be redundant [31].

With the optimization of a quantum edge sensor system via a versatile simulator, quantum edge sensors have the potential to outperform their classical counterparts. For example, this could generate chemical information on a compound detected within the sensor and local processor’s noisy environment with unprecedented sensitivity and fidelity. Given the lingering challenges and bottlenecks in chemical sensing [32], such an outcome would be of immense value. To realistically achieve such outcomes, early proposals will likely concentrate on a *classical* simulator of a purpose-built EQC and sensor network. This pilot simulator could then help the development of a more advanced simulator for designing quantum IoT (QIoT) applications or the development of a hybrid or all-quantum (analog or digital) simulator to serve the science and technology communities, potentially as part of national or international user facilities. A fully developed EQC simulator is expected to require HPC resources, allowing the sophisticated design of the future sensors, and the QIoT. Furthermore, building security (models and simulation capabilities) into what will initially be rare, shared quantum resources will be of colossal importance. Embedding threat models, attack taxonomy, and mitigation strategies, while ongoing for classical simulators [33], will be a critical element for any EQC simulator.

We will briefly discuss the objectives for the development of the EQC in Section 2, motivating factors in Section 3, and a research strategy in Section 4. We will then make concluding remarks in Section 5.

## 2. Objective

Our objective is to solicit a reaction from the community on the presented concept rather than to propose specific development directions. The latter, with literally every quantum subfield (as discussed here) being in its infancy, will be a monumental undertaking. Any proposal for the development of an EQC simulator requires defining and sequencing the simulation steps. One could begin by considering the basic characteristics of an edge device. To accomplish the goals of an *edge-native* simulator—as opposed to other compute architecture simulators such as the Structural Simulation Toolkit (SST) for HPC simulation [34], we must exactly define edge computing classically and quantum mechanically. The development of the SST required years of multi-institutional efforts (see [34] and the references therein). The result, an open, modular, parallel, multi-criteria, multi-scale simulation framework including a large library of memory, processor, network, and other models, can now be used for designing and procuring HPC systems. Undoubtedly, the same feat for EQC would require tremendous effort.

As is often the case with quantum phenomena, a quantum calculation or simulation ought to be able to reduce to the classical results or reproduce them. As such, a versatile EQC ought to be able to also function with classical elements. Recognizing that we need a simulation tool that supports the collective modeling of data generated by quantum sensing, computing, and networking resources, a logical objective may be to explore and address the following questions and associated challenges:

- What are the unique characteristics of quantum sensors to qualify as edge sensors? Are the characteristics generalizable to encompass classical sensors?
- What are the implications of sensor intelligence, which can vary as a function of distance (from the sensor to the user)? For example, how different cyber and cyber physical security measures should be implemented for a zero-intelligence sensor (one that generates only raw data) versus an intelligent sensor (one that is capable of some processing)?
- How do we model spatial and temporal variation (e.g., mobility) of the quantum sensors that impact qubit travel or mobility parameters, including latency and energy consumption?
- Is there a need for unifying the different quantum computing platforms? In an ideal scenario, one may envision a drag and drop of pre-defined quantum processor architectures at various locations of the network.
- How do we distribute tasks/loads, decompose algorithms, and allocate resources across multiple edge nodes?
- What specific resources and edge-native algorithms are needed to devise an EQC simulator?
- What are the unique features of an EQC system that classical counterparts lack?
- With enhanced quantum processing and communications, can there be collective effects that could be observed across multiple EQC nodes? If so, what models can be incorporated to account for such information?
- What other simulation-salient system properties and parameters are needed?

Answering these key research questions could generate the rigorous requirements and protocols needed to develop either a classical or quantum simulator for designing EQC and associated use cases. Given that quantum simulators are still in their infancy, a classical simulator running on HPC resources seems more rational. However, while classical simulators (e.g., RECAP [35]) may be useful for designing some ECC systems, no *edge-native* simulator has yet been reported [20].

## 3. Motivation

Quantum sensors [36] and quantum networks [8] are two hallmarks of the increasingly in-demand field of quantum science and technology [37] envisioned to enable new eras of metrology [36] and data use. As practical quantum hardware and software emerge [38] and the IoT grows, the integration of quantum sensors and quantum networks seems inevitable. The existing (classical) Internet and its IoT devices, guided by the available simulators, leverage ECC capabilities and sensors. Similarly, the future quantum internet [7] and its QIoT will leverage EQC and quantum sensors and will require a simulator. However, how such a simulator should function and be developed is unknown. Experience shows that classical tools sometimes do not carry over seamlessly to the quantum world—error correction is one example [39]. The development of a minimal classical pilot simulator for EQC and quantum sensors is therefore prudent. Such early work is a key element of co-design for a future science infrastructure consisting of QEC devices: sensors, processors, storage, compression, and communication, which in turn will enable new QIoT services. The recent development of quantum sensors, quantum computing, and quantum communications expertise worldwide may be leveraged to emulate a quantum edge device (sensor + processor). Relevant investigations in support of a need to address the objectives presented here are underway. For example, the characteristic features of edge devices are low power consumption, high processing power, and large memory capacities. These features are needed to carry out advanced machine learning at the edge, as discussed by Sludds et al. [40]. To counter the lack of the needed edge resources, Sludds et al. devised a method (still using the resource-constrained edge devices) to process data across networks and reported teraflops (10^12^ floating point operations per second or FLOPS) computing rates capitalizing on energy efficiency and sensitivity of photonics devices.

As a more basic example, let’s consider a specific EQC use case of environmental monitoring, e.g., the all-important chemical sensor [3]. Let this sensor leverage quantum mechanics to interrogate its environment for the presence of a specific compound of interest. The output data from the sensor may be in the form of its quantum state, such as the state of several qubits, or be presented classically, such as an output voltage. To process the data generated by this sensor, we provide quantum computing, for example via an ion-trap processor. Both the chemical sensor and the ion-trap processor constitute ideal candidates to qualify as a noisy intermediate-scale quantum (NISQ) system, since both the sensor and ion trap are highly susceptible to environmental noise, and the number of qubits is low. The processed data are either communicated back to the sensor, e.g., to control its subsystems, or transmitted to other EQC nodes or data centers.

If a simulator is to provide useful results for various EQC scenarios, the sensor(s) must be amenable to characterization using a standardized set of parameters. Investigating how such parameters can be represented in a logical, systematic, query-based quantum search or database platform is needed, especially if other emerging quantum sensing technologies or quantum processors are to be simulated, e.g., the explorative Majorana fermions [41,42] or topological materials [3]. Since the EQC nodes and devices are to communicate with each other, and the sensors (and nodes) may be spatially distributed and in a state of mixed deterministic and random motion, what quantum dynamic models, orchestration tools, and optimization strategies are of relevance for inter-communication? For example, at a classical (non-quantum) edge, the nomadic model [25] is often employed for movements. In the US alone, many research labs are addressing related questions. For example, computing resources at Oak Ridge National Laboratory, a US department of energy research facility hosting the world’s first exascale (>10^18^ FLOPS) supercomputer, Frontier [1], may be leveraged for use cases to be simulated. As it is expected that edge/IoT devices will number in the trillions over the next couple of decades [3], it is unclear if it is possible to use them in a coherent way. Can Frontier-scale data processing power be found in each of the edge devices as EQC evolves? Armed with artificial intelligence, quantum computing, quantum networking, and EQC, we will enter a completely new era of simulation.

## 4. Research Strategy

The strategy to develop a unified framework that allows a simulator to act in concert with diverse processing platforms may utilize the input–output relations for the individual (sub)system quantum components. Through this process, it may be possible to develop the framework for an EQC sensor-computing node. Undoubtedly, networked edge devices will exhibit many complex patterns. The resemblance of a universal network to self-similar topologies is evidence of the enormous complexity of the future smart world where the definition of the edge becomes increasingly relative. Here, to trace where and when quantum events occur, one may have to limit the spatial extent of the simulation to a smaller area. With reference to the figure above, the following quantum edge use case may be simulated: (1) the quantum edge sensor detects the (example) organic compound RDX (Tetranitro-triazacyclohexane) from a noisy environment; (2) the raw quantum data are properly presented and is stored in a quantum memory or communicated via a noisy quantum channel to a nearby ion-trap processor; (3) the processor will apply quantum machine learning [43,44] to discern and identify the spectral fingerprint of RDX; (4) if the processing capacity of the processor is depleted, the remaining processing task will be delegated [45] to another EQC node or to a quantum cloud; and (5) the final information distilled will be communicated to other EQC nodes and/or to a quantum cloud. The simulator must therefore cope with the needed processing power, processing speed, and architectural flexibility as classical bits and qubits will be processed interchangeably. The native ability of an EQC subsystem to accept input quantum states and output quantum states, as well as perform quantum computation, sets it apart from any current ECC simulators. Just as conventional supercomputers are incapable of simulating larger quantum (many-body) systems, ECC simulators may not be adequate for simulating quantum edge use cases, especially those involving a higher number of qubits.

To simulate the characteristic heterogeneity in edge sensors, several distinct quantum sensing platforms may be considered, for example, quantum imaging, quantum plasmonics, quantum photonics, and quantum-enhanced sensing using micro- and nano-electro-mechanical systems (MEMS and NEMS). The tremendous growth in mobile sensors and devices has delayed the development of much-needed “edge-native” simulators. What has appeared in the literature as a classical cloud-based simulator for commercial applications is not an edge-native simulator [3]. The research commented on here is instrumental for the co-design of quantum edge computing and sensing for future science and technology infrastructures. Any attempt to develop an EQC simulator will undoubtedly refine our understanding of quantum technologies and generate new IT venues.

## 5. Conclusions

From the presented brief discussion of an EQC simulator concept, significant efforts are needed to robustly define and standardize the initial set of classical and quantum elements that will make up the simulator components. Given the diversity of sensors and heterogenous output data types, processors, and computing tasks/loads, a modular approach to the components seems logical. Clearly, a simulator for quantum devices operating at the edge of a quantum network is expected to generate design and performance parameters that aid in the introduction of new edge devices or performance improvements of existing devices. These parameters are expected to guide the development of EQC devices that exhibit minimal power consumption, maximal local processing power, maximal data storage capacity, and minimal communication latency. Given the tremendous diversity of future sensors and networks applications and services, such development is not without analogy to the early development stage of the Internet, where the dissemination of the many HTML design software frameworks has allowed the creation of all the existing HTML web pages—the Internet currently hosts about 2 billion websites with the indexed web containing at least 4.24 billion pages [46]. By 2030, the expected number of connected IoT devices is to reach ~500 billion [47] with sensors being a crucial component. The number and innovativeness of edge devices providing future services will similarly depend on enabling co-design through simulators. The outlined basic example of a quantum edge use case, albeit lacking technical rigor or mathematical formulation, already exposes some of the tremendous challenges anticipated. This work is being reported with the anticipation that more insight can be garnered toward establishing a roadmap for EQC research.

## Data Availability

Not applicable.
